# Harnessing gastrointestinal motility for advancing diagnostics and therapeutics

**DOI:** 10.1016/j.treopn.2026.09.002

**Published:** 2026-10-06

**Authors:** Shonit N. Sharma, Akash A. Pai, Noah J. Selda, Xiangang Huang, Nimrat Kaur, Wei Tao, Jeffrey M. Karp, Yuhan Lee

**Affiliations:** 1 Department of Biological Engineering, Massachusetts Institute of Technology, Cambridge, MA, USA; 2 Center for Accelerated Medical Innovation, Department of Anesthesiology, Perioperative, and Pain Medicine, Brigham and Women’s Hospital, Boston, MA, USA; 3 Center for Nanomedicine, Department of Anesthesiology, Perioperative, and Pain Medicine, Brigham and Women’s Hospital, Boston, MA, USA; 4 Harvard Medical School, Boston, MA, USA; 5 Harvard–MIT Program in Health Sciences and Technology, Cambridge, MA, USA; 6 Harvard Stem Cell Institute, Cambridge, MA, USA; 7 Broad Institute of MIT and Harvard, Cambridge, MA, USA

## Abstract

Gastrointestinal (GI) motility plays a fundamental role in digestion, metabolism, immunity, and drug absorption. Yet, many current diagnostic and therapeutic devices designed for the GI tract overlook its dynamic mechanics, compromising their effectiveness and reliability. In this review, we provide a comprehensive overview of GI motility metrics, current devices and their limitations, and how a deeper understanding of motility can improve device design. We also highlight sex-specific differences in GI motility and their implications for clinical translation. We offer a perspective for a new approach that embeds standardized motility metrics into device development, thus improving diagnostic accuracy and personalizing therapies. By emphasizing physiology-guided design principles, future ingestible devices could adapt in real time to individual motility patterns, thereby transforming patient care.

## Gastrointestinal motility as a design principle for ingestible devices

The gastrointestinal (GI) tract is a complex biological system governed by multiple physiological processes—including pH regulation [1–3], enzymatic activity, and **motility** (see Glossary) [4,5]—which play important roles in digestion [6], absorption [7], and the overall health of the gut and body. Recent advancements in medicine and engineering have led to the development of devices that leverage the GI tract’s characteristics to diagnose [8], monitor [9], and treat diseases. Innovations such as **enteric-coated** capsules and **ingestible biosensors** [10] have emerged from manipulating pH and enzymatic functions. However, the potential to utilize GI motility remains largely untapped despite its significant role in physiological and pathological processes. Efficient gut motility is crucial for nutrient absorption, metabolic regulation, waste elimination, microbiome composition, immune function, and gut–brain signaling [11–20]. Abnormalities in motility are linked to metabolic disorders, inflammatory conditions, and several esophageal, gastric, and intestinal disorders, including those classified by the **Rome IV criteria** [21–32]. Motility disturbances can also arise following surgical and medical interventions, including cancer treatments and opioid use [33–36]. Although motility is central to GI diseases and treatment response, its influence on diagnostic and therapeutic device performance remains poorly understood. As motility is difficult to characterize and varies across individuals, disease states, and sex-specific physiology, it creates major challenges for the clinical translation of devices and highlights the need for more precise, individualized designs.

In this review, we comprehensively examine GI motility metrics and their implications for diagnostic and therapeutic applications, highlighting both challenges and opportunities that, to our knowledge, have not been previously addressed in the literature. After reviewing all metrics, to understand the limitations and capabilities of current devices, we explore a range of exemplary devices, from those routinely used in the clinic to those at the forefront of research. Accordingly, we emphasize the need for a more sophisticated approach to device design and function that can fully utilize the unique potential of the GI tract for improved health outcomes. In diagnostics, the analysis of GI motility patterns could lead to breakthroughs in early disease detection and a more profound understanding of digestive health, thereby facilitating personalized medical approaches. In therapeutics, we argue that the application of the GI tract’s inherent forces stands to revolutionize drug delivery-enhancing efficacy, reducing side effects through targeted therapy, and enabling real-time monitoring of treatment response. This approach could lead to novel drug formulations that are more personalized and efficient, significantly advancing patient care. Through collaborative efforts across disciplines, a thorough understanding of GI motility may enable us to strategically harness the dynamic environment of the GI tract—a burgeoning, transformative frontier—for improved diagnostics and therapeutics.

## Metrics can provide insights into harnessing the GI tract

The complex physiology of the GI tract shapes essential processes, including nutrient absorption, digestion, and **transit** of ingested matter (Figure 1A). Motility in the stomach and small intestine can be characterized by metrics that describe **contraction force**, **contraction frequency**, velocity, pressure, transit, and interactions with the luminal wall (Figure 1B,C). These metrics vary by GI region, prandial state, circadian rhythm, disease state, and measurement technique and should therefore be interpreted as approximate rather than universal.

### Motility is experienced and induced by ingested matter

Ingested matter (e.g., food and pills) is subjected to **intraluminal pressure** within the GI tract. Baseline pressure varies with posture, while the pressure required to initiate persistent propulsive contractions, caused by food or gas, differs across regions of the small intestine (Figure 1C) [43]. These regional differences reflect the heterogeneous mechanical environment of the intestinal lumen.

The relationship between motility and luminal contents is bidirectional. Ingested matter can stimulate contractions through stretch receptor activation [52], while the resulting contractions expose luminal contents to forces that vary with location and physiologic state. Gastric forces acting on pills, for example, differ substantially between fasted and fed conditions, and they increase during **gastric emptying** [37]. In the small intestine, circular and longitudinal smooth muscle also generate different forces [51]. Together, these observations illustrate how luminal contents both experience and influence GI mechanical activity, which is critical for designing ingestible systems that can withstand, respond to, or harness luminal forces to enable controlled release and reliable **actuation** [37,38,44,53].

### Stomach motility metrics are influenced by feeding conditions

Gastric motility can be characterized by contraction frequency, velocity, force, and emptying behavior (Figure 1B) [39]. The antrum exhibits distinct contraction frequency, **propagation velocity**, and grinding forces, reflecting its important role in mixing and mechanically processing gastric contents [40].

Meal properties further modify these dynamics. Increasing viscosity delays gastric emptying, and liquids and solids experience different cumulative forces during processing [40]. Gastric force amplitude and the forces associated with emptying also vary with **prandial state** [38]. Feeding similarly alters the chemical environment, including gastric pH [41]. Collectively, these findings demonstrate that gastric motility is highly dependent on both the physical properties of ingested matter and feeding conditions.

### Small intestine motility metrics are important for drug absorption

The small intestine is a major site of nutrient and drug absorption and is mechanically active throughout transit. Its contractions can be characterized by force amplitude, frequency, velocity, diameter change, and **radial force** (Figure 1C) [38,39,44,46,47]. Propagating contractions range from normal **peristalsis** to much faster rush peristalsis [39]. These mechanical properties are also relevant to drug formulation behavior, including tablet hardness and drug release [53]. Human measurements of small intestine motility remain comparatively limited, so studies in anatomically or physiologically similar species can supplement human observations. In dogs, small intestinal transit is shorter than in humans, with reported transit times of approximately 1.6 to 3.7 h [48,49]. Pigs share several anatomical features with the human small intestine, including a similar intestinal length-to-body-weight ratio of approximately 0.1 m/kg and a comparable luminal diameter, supporting their use as translational models of GI function [54]. Together, these interspecies data help fill gaps in human small intestine metrics and support the use of anatomically and physiologically similar animal models for *in vivo* studies aimed at understanding GI function and improving human-relevant diagnostics and interventions while recognizing species-specific differences in motility.

### Transit and wall contact metrics help explain drug absorption profiles

Transit and **wall contact** jointly govern how ingested matter moves through the GI tract. Gastric chyme can move rapidly through the pyloric region, exerting pressure on food particles [42]. In the small intestine, the velocity of ingested matter varies with the feeding state, while contractions occur over characteristic durations [50]. Transit is similarly state-dependent, as demonstrated by differences in the passage of non-dissolving tablets under fasted and fed conditions [45].

These transport dynamics are coupled to interactions with the intestinal wall. **Mucoseparation energy** and coefficient of friction characterize adhesion and sliding at the mucosal interface, while shear angle describes regional deformation of the intestinal wall [44]. Together, propagation, transit, and wall interaction determine the **residence time**, contact, and mechanical experience of ingested matter as it moves through the intestine.

### Sex-based differences influence GI motility metrics

There is significant variation in GI motility metrics between females and males. Females generally exhibit slower gastric emptying times, small intestinal transit times, and colonic transit times than males. Hormonal variations, especially estrogen and progesterone, contribute to these differences by inhibiting gastric motility [55,56]. A scintigraphy study showed that females have a significantly slower post-lag gastric emptying rate than males [57]. Another study found that females have a longer half-emptying time for both solids and liquids than males. The solid half-emptying time for females was 92.4 ± 7.5 min, while for males it was 59.8 ± 3.7 min, and the liquid half-emptying time for females was 53.8 ± 4.9 min, while for males it was 30.3 ± 2.3 min [58]. This may, in part, be attributed to hormone levels—gastric emptying slows when ovarian hormone levels are high, and estrogen and progesterone are inhibitors of smooth muscle contraction [58]. Additionally, the menstrual cycle affects gastric motility, although studies have reported variable phase-specific effects on gastric emptying [56]. These differences in motility are crucial for understanding sex-specific responses to GI treatments and interventions.

### Drugs affect metrics by stimulating or relaxing GI motility

Drugs play an important role in modulating motility metrics (e.g., intraluminal pressure) and the function of the GI tract as a whole [59,60]. Calcium channel blockers, nitrates, phosphodiesterase type 5 inhibitors, and botulinum toxin can relax esophageal smooth muscle, whereas **prokinetic agents** such as sincalide, metoclopramide, domperidone, and erythromycin can stimulate GI contractions [61,62]. Motility is also regulated by the enteric and autonomic nervous systems and by local and circulating mediators, including ghrelin, cholecystokinin, motilin, glucagon-like peptide-1, serotonin, and dopamine [63,64]. These components of the ‘gut–brain’ are susceptible to therapeutic modulation through opioid, adrenergic, cholinergic, prostaglandin, and related pathways [65,66]. However, drug effects on motility can be variable, and agents such as anti-emetics may differ in efficacy across patients [67].

Studies in animals and humans further show that metoclopramide, bethanechol, and domperidone can increase esophageal peristaltic activity, esophageal tone, and gastric motor activity [62]. Physiologic state also affects measured motility: in dogs, anesthesia reduced gastric maximum and mean intraluminal pressures from 6.9 and 1.5 kPa to 1.1 and 0.4 kPa, respectively, and small intestinal pressures from 10.0 and 0.5 kPa to 1.7 and 0.4 kPa [43,68]. These results suggest that there is a marked reduction in contraction strength postanesthesia. Together, these findings underscore the complex interplay between therapeutics and motility, emphasizing the importance of understanding motility metrics for effective diagnostic and therapeutic development.

### Disease effects on metrics can be localized to specific organs

Pathological changes in different organs are a significant factor causing variation in motility metrics (e.g., transit time). We note several diseases that affect motility throughout the GI tract, from the esophagus to the large intestine (Box 1).

### Laboratory simulations of the GI tract can aid our understanding of motility

*In vitro* and *in silico* models are used to study GI contractions, providing key insights into human processes (Box 2).

## Current GI devices demonstrate opportunities and limitations in a dynamic environment

Current diagnostic and therapeutic GI technologies demonstrate both the clinical value of measuring or interacting with motility and the limitations of devices that remain dependent on native, variable GI forces (Table 1). Non-ingestible diagnostic platforms, including magnetic resonance imaging (MRI), high-resolution **manometry**, and body-surface gastric mapping, can characterize motion, pressure, and electrical activity with increasing spatial and temporal resolution. However, these approaches remain limited by invasiveness, episodic sampling, patient tolerance, procedural complexity, or indirect measurement of mechanical forces. Ingestible diagnostic systems, including capsule endoscopy and electromagnetic capsule tracking, improve comfort and ambulatory monitoring, but their performance depends on unpredictable transit, residence time, wall contact, and regional motility patterns.

Therapeutic GI devices face similar challenges. Ingestible **milli-injectors**, including spring-driven, gas-generating, and unfolding microneedle capsules, have shown that physical placement of biologics into GI tissue can improve oral delivery of macromolecules. Yet these systems often depend on stable wall contact, correct orientation, regional localization, and timely actuation within a deformable and actively contracting lumen. Non-ingestible therapeutic implants, including sphincter augmentation, gastric restriction, and artificial sphincter devices, can restore or modify GI function, but fixed mechanical set points may not adapt to prandial state, posture, reflex activity, or patient-specific motility. Across these examples, the central engineering challenge is that motility directly shapes sensing reliability, device localization, actuation timing, retention, and therapeutic delivery.

## Harnessing motility can improve ingestible diagnostics and therapeutics

Next-generation GI devices could improve reliability by sensing, adapting to, or actively harnessing motility rather than passively tolerating it (Figure 2A). For diagnostics, motility-aware capsules could use local pressure, velocity, pH, orientation, or contact signals to adjust image capture, sampling, retention, or transit in real time (Figure 2B). Coupling these measurements with computational modeling, including **computational fluid dynamics** software such as COMSOL Multiphysics, to assess the stability of devices as they flow through the GI tract, or **artificial intelligence (AI)**-based interpretation could help predict regional transit, identify abnormal contraction patterns, and trigger data collection during physiologically informative windows (Box 3). Adhesive or anchoring interfaces could further extend mucosal contact when prolonged monitoring is needed, provided that adhesion is reversible and matched to local shear and contraction forces.

For therapeutics, motility can be treated as an endogenous source of mechanical energy (Figure 2C). Instead of relying solely on stored springs, balloons, or single-shot deployment mechanisms, drug delivery capsules could use peristaltic compression, intraluminal pressure, or cyclic wall contact to trigger release, improve tissue apposition, or enable repeated dosing. Such designs may reduce the risk of failure from mistimed actuation and improve delivery across varying feeding states and transit patterns. More broadly, motility-informed design reframes the GI tract as an active mechanical partner for sensing, actuation, retention, and localized therapy rather than as a hostile and unpredictable environment.

Motility-informed personalization could further improve ingestible diagnostics and therapeutics for an individual’s anatomy, physiology, and disease state. Although comprehensive measurement of GI metrics would be ideal, available data remain uneven across GI regions, measurement methods, and patient populations. Important gaps remain regarding demographic variables such as age, sex, and race, and the literature linking genetic biomarkers to GI motility is especially limited. Even so, existing motility metrics, anatomical data, and current device designs provide a foundation for next-generation personalized and **closed-loop** systems.

Sex-based differences in GI motility are rarely incorporated into ingestible device design, despite their effects on transit time, residence time, wall contact, and actuation windows. As mentioned, females have been reported to have gastric half-emptying times approximately 55% longer for solids and 78% longer for liquids than those of males [58]. These differences suggest that capsules designed for prolonged regional residence may require different retention-enhancing features depending on patient physiology. For instance, a device intended for populations with faster transit could incorporate stronger or longer-lasting adhesion, such as a thicker mucoadhesive coating, to achieve an observation or delivery window comparable to that of a device with weaker adhesion in populations with slower transit. Sex-informed actuation thresholds could also help ensure that diagnostic sampling or therapeutic release occurs during an appropriate motility window rather than at a fixed time point. Integrating sex-specific motility data into GI device design could improve reliability, personalization, and equity.

One potential closed-loop system is an ingestible device for gastroparesis that combines motility sensing with triggered drug release (Figure 2D). Such a capsule could use an accelerometer to detect delayed gastric movement relative to the individual’s established baseline motility pattern, while a biochemical sensor monitors prolonged gastric acidity as an additional marker of delayed emptying [128]. If both motility and pH criteria are met, the device could trigger release of a prokinetic agent such as prucalopride or erythromycin through a gas-generating or pressure-responsive mechanism. This dual-validation strategy could enable on-demand therapy while reducing unnecessary drug exposure.

Translation of motility-informed systems will require not only improved access to GI metric datasets but also prospective clinical studies that test device behavior under realistic physiological conditions. Key endpoints may include localization accuracy, residence time, tissue contact, actuation success, dose delivery, diagnostic yield, patient comfort, and adverse events. These studies should evaluate performance across prandial and disease states, medication exposures, and demographic variables, including age, sex, and race. Devices that are highly personalized but too complex, costly, or difficult to manufacture may fail to deliver meaningful clinical benefit. Progress will, therefore, depend on multidisciplinary collaboration to develop systems that are simple, scalable, sustainable, and clinically practical. Working with GI motility, rather than trying to circumvent it, may enable simpler and more reliable ingestible diagnostic and therapeutic devices.

## Concluding remarks

The GI tract, an intricate physiological system and a marvel of biological engineering, offers untapped opportunities to revolutionize diagnostics and therapeutics. However, engineering within the GI space is constrained by limited data on key motility metrics. By advancing our understanding of its dynamic motility, we can unlock new methods for diagnosing and treating diseases. Harnessing GI motility in conjunction with real-time, patient-specific physiological data could enable the development of ingestible devices that adapt to the unique rhythms of each individual, significantly improving the precision and personalization of care. Incorporating sex-specific data into device design allows for the development of tools that not only adapt to individual motility patterns but also account for physiological variations across different populations. Such innovations will not only enhance the reliability of diagnostic devices but also pave the way for targeted drug delivery systems and more effective therapeutic interventions, advancing more equitable healthcare solutions and transforming the future of medicine. Answering the outstanding questions (see Outstanding questions) and achieving this vision both require interdisciplinary collaboration and the integration of motility metrics into a physiology-guided approach to medical device design, offering a path toward more personalized, inclusive, and effective healthcare solutions. Going forward, closed-loop, motility-harnessing ingestible devices capable of measuring GI metrics in real time and modulating their behavior in real time represent a promising paradigm for personalized GI diagnostics and therapeutics.

## Figures and Tables

**Figure 1.**
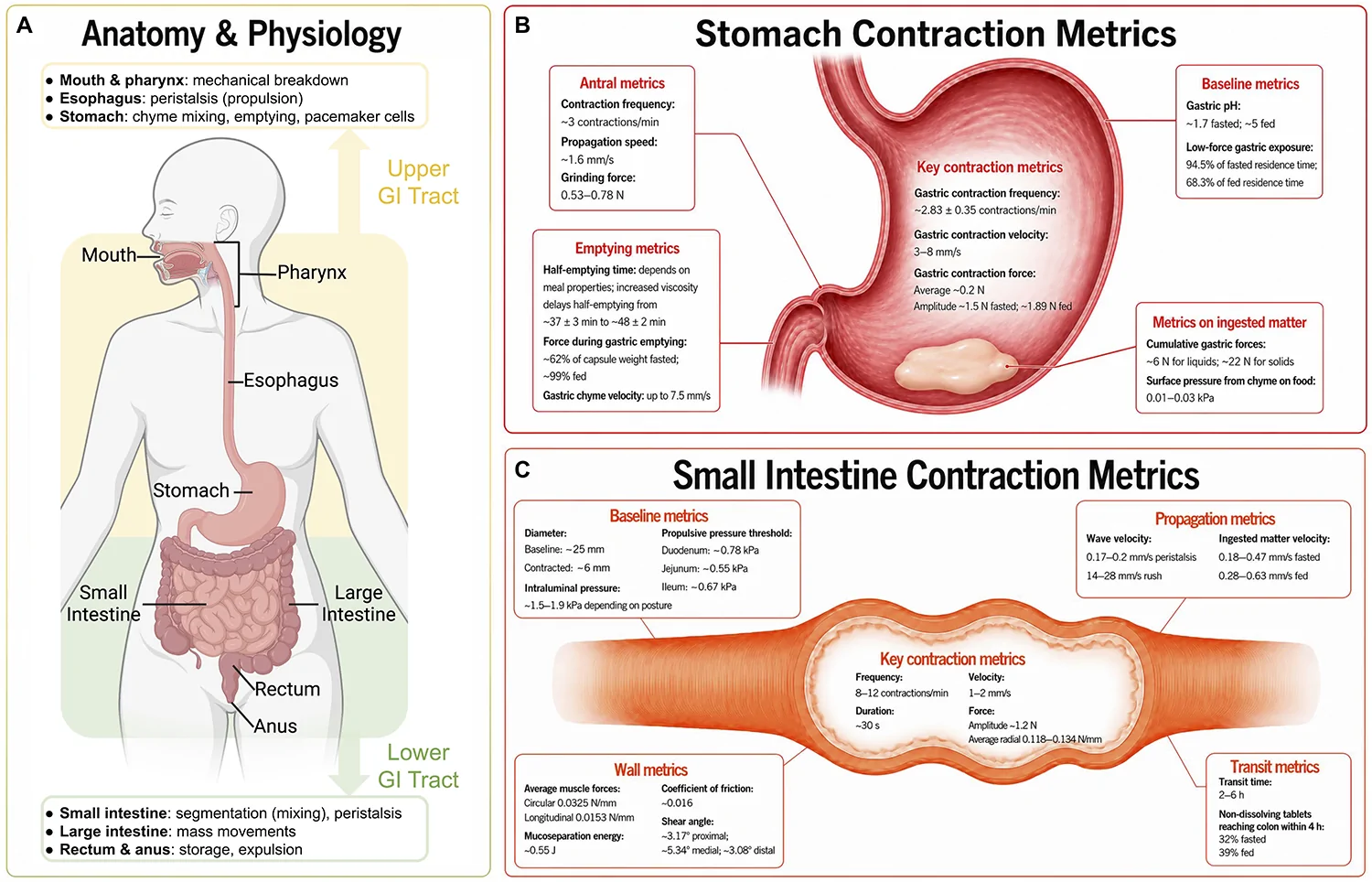
Gastrointestinal (GI) tract anatomy and physiology influence contraction metrics. (A) Anatomy and physiology of the upper and lower GI tracts, highlighting major regions and motility patterns involved in the movement and processing of ingested matter. (B) Quantitative summary of stomach motility metrics reported in the literature, including contraction frequency, velocity, force, gastric emptying, pH, and forces experienced by ingested matter under fasted and fed conditions [37–42]. (C) Quantitative summary of small intestine motility metrics, including contraction frequency, velocity, force, intraluminal pressure, diameter, propagation, transit, and wall interaction properties [38,39,43–51]. Created with BioRender (www.biorender.com).

**Figure 2.**
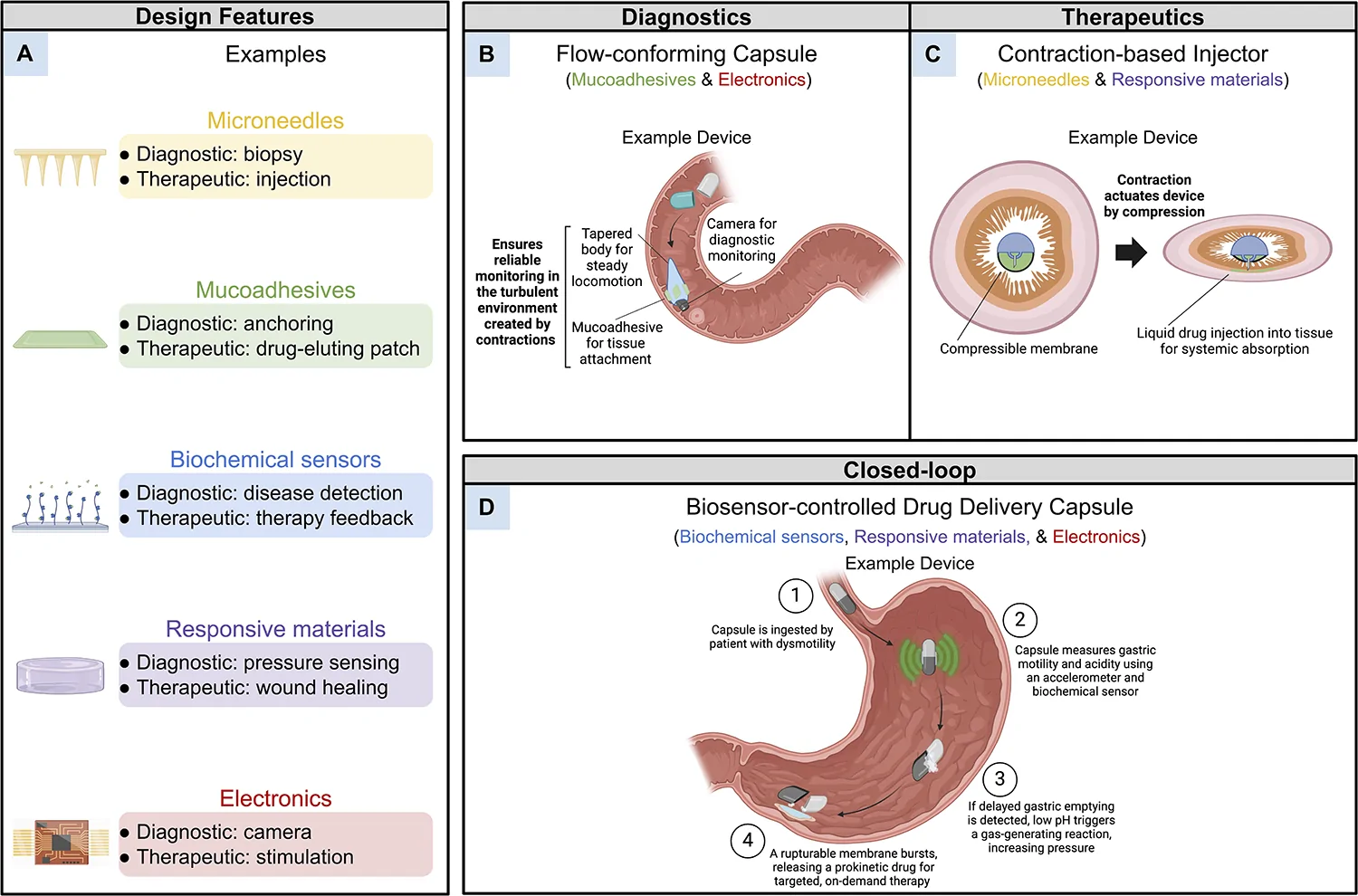
Motility can be harnessed as a design input for next-generation gastrointestinal (GI) diagnostic and therapeutic devices. (A) Design features that utilize motility can introduce beneficial diagnostic and therapeutic functionalities to GI devices. (B) A flow-conforming capsule is an example of an ingestible diagnostic device that could harness motility to ensure reliable monitoring in the turbulent environment created by contractions in the GI tract. (C) A contraction-based injector is an example of an ingestible therapeutic device that could harness motility to ensure device actuation in the form of liquid drug injection into GI tissue for systemic absorption. (D) A biosensor-controlled drug delivery capsule is an example of a closed-loop device that could integrate accelerometer-based motility sensing to detect delayed gastric emptying and subsequently use a biochemical sensor to verify prolonged gastric acidity, triggering a gas-generating reaction that ruptures a membrane for on-demand prokinetic drug release. Created with BioRender (www.biorender.com).

**Table 1.** Gastrointestinal motility imposes device-specific constraints that can be harnessed to improve diagnostic and therapeutic performance

Device	Actuation mechanism and function	Governing motility metrics (shown in Figure 1)	Engineering constraint imposed by motility	Motility-harnessing opportunity	References
**Ingestible diagnostics**					
Capsule endoscopy (e.g., PillCam SB3)	Camera-based mucosal imaging during passive transit through the GI tract	Small intestinal contraction frequency; small intestinal contraction velocity; small intestinal peristaltic wave velocity; small intestinal rush wave velocity; small intestinal transit time; colon arrival time; small intestinal radial force	Native peristalsis dictates capsule motion, so rapid transit can miss regions of interest, delayed transit can limit recording time, and variable residence time can produce incomplete or poorly timed imaging	Add local velocity sensing, adaptive image capture, transient braking, micro-actuation, or anchoring to synchronize imaging with motility patterns and improve regional coverage	[38, 42, 45, 48, 91–98]
Electromagnetic capsule tracking (e.g., 3D-Transit system)	Ingestible electromagnetic capsule tracked by a wearable sensor belt to map position and orientation during ambulatory transit	Gastric chyme velocity; gastric surface pressure from chyme on food; small intestinal ingested matter velocity; small intestinal peristaltic contraction duration; colon arrival time	Capsule displacement is an indirect proxy for contraction, and posture, belt motion, feeding state, or external movement can affect kinematic measurements	Fuse positional tracking with onboard pressure, inertial, or contact sensing to distinguish passive displacement from contraction-driven movement	[45, 51, 52, 99]
**Non-ingestible diagnostics**					
Magnetic resonance imaging (e.g., MRI, cine-MRI, functional MRI)	Noninvasive imaging used to visualize and quantify GI motion without radiation	Baseline small intestinal diameter; contracted small intestinal diameter; small intestinal contraction velocity; small intestinal peristaltic wave velocity; small intestinal rush wave velocity; gastric contraction velocity; small intestinal transit time	Imaging can quantify motion but may be resource-intensive, episodic, and dependent on analytic methods for extracting complex motility patterns	Use imaging-derived motility maps to define device size, deformation tolerance, retention requirements, actuation windows, and expected regional transit behavior	[42, 47, 100]
High-resolution manometry (e.g., fiber optic manometry)	Pressure-sensor catheter measures luminal pressure patterns, contraction amplitude, propagation, and sphincter function	Small intestinal intraluminal pressure; small intestinal propulsive pressure threshold	Catheter placement, anesthesia, posture, and recording conditions can alter the motility being measured; patient tolerance limits recording duration, and interpretation requires specialized protocols and expertise	Miniaturize pressure sensors, reduce tethering, use contraction-force feedback for positioning, or develop capsule-based pressure mapping as a comfort-preserving alternative	[37, 68, 91, 92, 101–111]
Body-surface gastric mapping (e.g., silver/silver chloride (Ag/AgCl) electrodes)	Electrode arrays measure GI slow-wave activity, including rhythm stability, propagation direction, velocity, and amplitude	Gastric contraction frequency; gastric contraction velocity; antral contraction frequency; antral propagation speed	Electrical mapping captures rhythm and propagation but does not directly measure contractile force, wall contact, or drug/device transit	Pair electrical mapping with ingestible sensors or actuators to time sampling, stimulation, image capture, or drug release to physiological motility cycles	[42, 43, 112]
**Ingestible therapeutics**					
Spring-driven gastric milli-injector capsule (e.g., L-SOMA)	Fluid-triggered dissolution releases a spring-driven injection mechanism to deliver macromolecules or nanoparticles into the stomach wall	Gastric pH; gastric half-emptying time; gastric contraction frequency; gastric contraction velocity; gastric contraction force; antral contraction frequency; antral propagation speed; antral grinding force; low-force gastric exposure; force during gastric emptying	Single-shot actuation can fail if deployment occurs without stable wall contact or during displacement by gastric contractions; feeding state changes force exposure, pH, and emptying dynamics	Gate firing using pressure, orientation, or contact signatures; use native gastric force profiles to improve timing and reliability of tissue injection	[39, 40, 42–44, 113–117]
Gas-generating intestinal milli-injector capsule (e.g., RaniPill)	Enteric coating dissolves in the small intestine, enabling CO_2_ generation that inflates a balloon and drives a biodegradable microneedle into the intestinal wall	Gastric pH; baseline small intestinal diameter; contracted small intestinal diameter; small intestinal contraction frequency; small intestinal contraction force; small intestinal transit time; small intestinal radial force	pH-triggered actuation confirms regional entry but not necessarily stable wall contact; local contractions may alter contact time, force exposure, and injection orientation	Couple pH triggering with pressure, contact, or orientation sensing to ensure deployment during a stable tissue-contact window	[38, 40, 42, 44,46–48, 115, 116, 118]
Unfolding intestinal milli-injector capsule (e.g., LUMI)	Luminal unfolding microneedle injector deploys arms to position microneedles against GI tissue for macromolecule delivery	Baseline small intestinal diameter; contracted small intestinal diameter; small intestinal contraction frequency; small intestinal contraction velocity; small intestinal contraction force; small intestinal radial force; small intestinal mucoseparation energy; small intestinal coefficient of friction; small intestinal shear angle	Unfolding and microneedle placement must occur within a deformable lumen with variable diameter, shear, radial force, and wall contact	Use regional diameter, contraction, radial-force, and adhesion metrics to tune unfolding geometry, contact pressure, anchoring force, and retention during delivery	[38, 40, 42, 46, 47, 115, 116, 119]
Mucosal adhesion devices (e.g., electroadhesive hydrogel)	Mechanical interlocking or electroadhesion maintains mucosal contact for prolonged sensing or therapy	Baseline small intestinal diameter; contracted small intestinal diameter; small intestinal radial force; small intestinal contraction duration; small intestinal mucoseparation energy; small intestinal coefficient of friction; small intestinal shear angle	Adhesion must withstand cyclic shear, radial loading, and deformation without causing mucosal injury, obstruction, or unsafe retention	Develop switchable retention systems that adhere during high-force windows and release on command	[38, 47, 52, 120, 121]
Self-propelling ingestible device (e.g., INSPIRE)	Self-propelling device mechanically stimulates the bowel to promote intestinal transit	Small intestinal peristaltic wave velocity; small intestinal rush wave velocity; small intestinal ingested matter velocity; small intestinal circular muscle force; small intestinal longitudinal muscle force; small intestinal coefficient of friction	The device must generate directional motion in a low-friction, compliant, actively contracting lumen where native propagation differs by feeding state	Exploit regional force anisotropy and cyclic wall contact to generate traction rather than resisting motility	[38, 42, 52, 54, 122]
Compressible capsule (e.g., RNACap)	Capsule releases liquid mRNA therapeutics when compressed by endogenous intestinal contractions	Baseline small intestinal diameter; contracted small intestinal diameter; small intestinal contraction frequency; small intestinal contraction velocity; small intestinal contraction duration; small intestinal contraction force; small intestinal radial force	Delivery depends on receiving sufficient compressive events, so dose release may vary with patient-specific motility, feeding state, and regional force exposure	Use endogenous compression for repeated, energy-free actuation; tune capsule mechanics to match regional contraction forces and contraction duration	[38, 40, 42, 46, 47, 52, 123]
**Non-ingestible therapeutics**					
Magnetic sphincter augmentation (e.g., LINX Reflux Management System)	Magnetically coupled titanium beads augment lower esophageal sphincter closure and separate during swallowing	Esophageal peristaltic pressure; lower esophageal sphincter resting pressure; swallow opening pressure (metrics not shown in Figure 1)	Fixed magnetic resistance must balance reflux prevention with swallow-mediated opening; mismatch can contribute to dysphagia, erosion, or device removal	Develop pressure-responsive or actively modulated magnetic elements that adjust closing force to measured swallow dynamics	[37, 106, 124, 125]
Gastric restriction (e.g., adjustable gastric band)	Hydraulic restriction at the gastroesophageal junction delays gastric emptying and promotes satiety	Gastric pH; gastric half-emptying time; gastric contraction frequency; gastric contraction velocity; gastric contraction force; antral contraction frequency; antral propagation speed; antral grinding force; cumulative gastric forces	Constant restriction may not adapt to prandial, circadian, or disease-related changes in gastric motility, contributing to tissue adaptation, wear, or erosion	Use adjustable or closed-loop restriction guided by measured antral activity, meal state, gastric force, or gastric emptying dynamics	[40, 43, 44, 126]
Artificial sphincter (e.g., anal sphincter implant)	Mechanical, fluidic, or magnetic compression restores continence while preserving voluntary function	Anal resting pressure; anal squeeze pressure; rectal distension pressure; reflex-mediated anal relaxation (metrics not shown in Figure 1)	Constant occlusion may not adapt to rectal distension, reflex activity, tissue remodeling, posture-related pressure shifts, or patient-specific pressure requirements	Use closed-loop cuff pressure guided by sensed rectal distension, sphincter pressure, reflex activity, or posture-related pressure changes	[37, 106, 107, 127]

Representative ingestible and non-ingestible GI technologies are organized by device class, actuation mechanism and function, governing motility metrics, engineering constraints imposed by motility, and motility-harnessing opportunities as a guide for improving device performance [37–48,50,51,68,91–127]. Motility metric values are summarized in Figure 1 unless otherwise specified.

## Significance

The gastrointestinal tract constantly squeezes, mixes, and moves the contents we swallow. This complex process known as motility affects how ingestible devices—including cameras, sensors, and drug delivery capsules—travel, collect information, and release treatments, especially because motility differs across patients. This review explains why motility should guide device design by summarizing key gastrointestinal metrics, current device limitations, and opportunities to harness motility for more reliable, personalized diagnosis and therapy that ultimately improves patient care.

## Outstanding questions

Can purely mechanical transduction of GI contractile forces eliminate the need for onboard energy storage in ingestible therapeutic devices?

How variable are individual motility profiles over time, and can this intraindividual variability be captured with sufficient resolution to personalize device behavior?

What role can AI play in predicting GI motility patterns from limited sensor data to enable real-time adaptive device control?

How should cyclic hormonal influences on GI motility be accounted for in the design and dosing strategies of motility-responsive devices?

Can a single ingestible platform distinguish healthy from pathological contraction patterns *in situ*, enabling combined diagnostics and therapeutics?

What standardized *in vitro* and *in silico* benchmarks are needed to evaluate motility-responsive device performance before advancing to animal and human studies?

How can multi-actuation release mechanisms ensure complete payload delivery across the range of physiological conditions encountered in the GI tract?

## Box 1. Motility disorders across the GI tract

In the esophagus, diseases such as achalasia and hypercontractile esophagus are characterized by impaired relaxation or excessive contractions that disrupt normal swallowing [69–72]. These conditions are diagnosed using manometry and imaging, including a barium swallow study (esophagram), and are treated with interventions that directly modulate motility, including pneumatic dilation and peroral endoscopic myotomy, as well as pharmacologic therapies targeting smooth muscle tone.

In the stomach, gastroparesis is defined by delayed gastric emptying due to impaired or weakened contractions and is associated with conditions such as diabetes and neurological diseases [5,73,74]. Management strategies include prokinetic agents and gastric electrical stimulation, which aim to restore or augment motility. Cyclic vomiting syndrome reflects dysregulation of gut–brain interactions and gastric function and is managed through behavioral, pharmacologic, and supportive interventions [48,75].

In the small intestine, chronic intestinal pseudo-obstruction presents with symptoms resembling mechanical obstruction despite the absence of a physical blockage, arising from neuromuscular dysfunction that impairs coordinated transit [26,76]. Treatment focuses on stimulating motility and managing symptoms, often through pharmacologic therapies and, in severe cases, surgical or supportive interventions. In the large intestine, inflammatory bowel disease, affecting approximately 1.3% of US adults, is associated with altered motility patterns that both influence and are influenced by inflammation [77–79]. Management includes anti-inflammatory and immunosuppressive therapies alongside surgical interventions in advanced disease, which indirectly modulate motility by reducing the inflammatory burden.

Motility disturbances may arise from diverse mechanisms, including neuromuscular dysfunction, inflammation, metabolic disease, and external factors such as chemotherapy and other medical interventions. Across these diseases, diagnostic and therapeutic technologies, including manometry, imaging, electrical stimulation, and surgical or endoscopic interventions, can measure or directly manipulate motility. Together, these examples highlight that motility is both a target and a mediator of intervention, underscoring the need for technologies that can reliably function within, and ultimately harness, the dynamic environment of the GI tract.

## Box 2. Laboratory simulations of the GI tract

Laboratory-created artificial stomach or intestinal models, developed through interdisciplinary engineering techniques, offer an integrated *in vitro* platform for testing devices, medications, and other GI treatments [80]. By using real tissue or tissue-like material as a lining, along with soft robotics to mimic the contractile forces found in the GI tract, microcontrollers can be used to manage the strength and cycle frequency of the robotic system, ensuring accurate simulation of GI anatomy and physiology. Hydrogels, whether used alone or within artificial models, respond to external stimuli such as pressure, pH, or temperature changes [81], increasing the accuracy of the simulated environment. Tissue cultures or laboratory-sourced organoids can simulate the complex gastric environment or gastric contractions *in vitro* [82,83]. Cells derived from gastric stem cells can be cultured in an organ-specific environment and then formed into organoids that resemble the target tissue. These three-dimensional structures then mimic the physiological properties of the stomach or intestinal tract. Organoids can be subjected to mechanical and electrical stimulation to induce contractions. In this way, tissue cultures may accurately recapitulate the complex biology of the GI tract.

Microfluidic systems can be employed to replicate the complex process of contractions [84–86]. By managing the flow of fluids within microchannels, these devices imitate the movement of stomach contents and the effects of muscular contractions, using pumps to simulate peristaltic flow. Moreover, these devices enable the direct observation of fluid dynamics and contractions using microscopy or other imaging methods. For better visualization, substances such as fluorescent dyes or particles may be incorporated into the fluid, allowing for the tracking of flow and structural changes within the microchannels, thus offering precise measurements of contractions. In summary, microfluidic devices offer a controlled platform for studying GI motility, advancing diagnostic and therapeutic applications.

Computational simulations can be used in conjunction with electrical stimulation, whether direct or transcutaneous, which is a rapidly emerging field in modern precision medicine [87–89]. Neuronal activation around vagal afferents and the stomach has been shown to simulate the effect that an electrical pulse would have on gastric motility [90]. Complex simulations, often involving high-fidelity finite element models and active nerve models, are created to study the effectiveness of different electrical stimulation modalities in increasing gastric motility.

## Box 3. AI in GI motility diagnostics and therapeutics

AI could accelerate the development of motility-informed GI diagnostics and therapeutics by extracting clinically meaningful patterns from complex, multimodal data. In diagnostics, AI could assist with analysis of cine-MRI, functional MRI, high-resolution manometry, multielectrode recordings, gastric body-surface mapping, capsule endoscopy, and electromagnetic capsule tracking [91–112]. These approaches could help identify abnormal contraction patterns, predict regional transit, quantify residence time, and distinguish physiological variability from disease-associated motility changes [97].

AI may also support next-generation therapeutic devices by enabling real-time interpretation of pressure, pH, velocity, orientation, contact, and biochemical signals. These data could guide closed-loop decisions, including when to image, sample, anchor, actuate, or release a therapeutic payload [113–123]. However, AI-enabled GI devices will require standardized datasets, prospective validation, interpretable models, and inclusion of diverse patient populations across age, sex, race, disease state, medication use, and prandial state. Without these safeguards, AI could reinforce existing biases in motility data rather than improve personalized care.

## Glossary

Actuation: the process by which a device performs a function (e.g., releasing a drug, deploying a sensor, or changing shape).
Artificial intelligence (AI): computational methods that identify patterns in complex data. In GI motility, AI could help interpret imaging, pressure, electrical, or capsule-tracking signals.
Closed-loop: a device or system that senses physiological signals (e.g., pH and pressure) and automatically adjusts a function (e.g., drug release) in response.
Computational fluid dynamics: computer-based modeling of fluid flow and mechanical forces in dynamic environments.
Contraction force: mechanical force generated by GI smooth muscle during contractions, which can influence capsule displacement, tissue contact, stability, and actuation.
Contraction frequency: the number of GI contractions occurring over a defined time interval.
Enteric coating: a pH-responsive capsule or tablet coating typically designed to resist dissolution in the acidic stomach and release its contents in the more neutral small intestine.
Gastric emptying: the movement of stomach contents into the small intestine. Gastric emptying rate affects drug dissolution, capsule transit, nutrient absorption, and device residence time.
Ingestible biosensor: a swallowable device that measures physiological or biochemical signals within the GI tract (e.g., pH, temperature, pressure, enzymes, and biomarkers).
Intraluminal pressure: the pressure within the hollow space of the GI tract. It reflects contractile activity and can influence device movement, retention, actuation, and drug release.
Manometry: a diagnostic technique that measures pressure changes within the GI tract using pressure-sensitive sensors or catheters.
Milli-injectors: an ingestible drug delivery device that physically injects a therapeutic payload into GI tissue to improve systemic absorption.
Motility: coordinated contractions and relaxations of smooth muscle that move, mix, and retain contents within the GI tract.
Mucoseparation energy: the energy required to separate a material or device from the GI mucosal surface. It helps describe adhesion strength and retention.
Peristalsis: a coordinated wave of GI smooth muscle contraction that propels luminal contents forward.
Prandial state: the feeding condition of the GI tract, typically fasted or fed.
Prokinetic agent: a drug that stimulates GI motility to improve transit or emptying.
Propagation velocity: the speed at which a contraction wave or luminal contents move through a GI region.
Radial force: the force exerted outward or inward on the intestinal wall or a device surface during luminal deformation.
Residence time: the duration for which food, fluids, drugs, or devices remain within a specific GI region.
Rome IV criteria: a standardized framework for classifying disorders of gut–brain interaction, including functional GI disorders.
Transit: the process by which contents or devices move through part or all of the GI tract.
Wall contact: the physical interaction between a device and the GI mucosa, which can affect sensing, retention, injection, and localized drug delivery.
